# Structure-Function Elucidation of a New α-Conotoxin, MilIA, from *Conus milneedwardsi*

**DOI:** 10.3390/md17090535

**Published:** 2019-09-16

**Authors:** Steve Peigneur, Prabha Devi, Andrea Seldeslachts, Samuthirapandian Ravichandran, Loïc Quinton, Jan Tytgat

**Affiliations:** 1 Toxicology and Pharmacology, KU Leuven, Campus Gasthuisberg, O & N2, Herestraat 49, P.O. Box 922, 3000 Leuven, Belgium; steve.peigneur@pharm.kuleuven.be (S.P.); dprabha@nio.org (P.D.); andrea.seldeslachts@gmail.com (A.S.); 2 CSIR-National Institute of Oceanography, Dona Paula, Goa 403 004, India; 3 Center of Advanced Study in Marine Biology, Annamalai University, Parangipettai, Tamil Nadu 608 502, India; sravicas@gmail.com; 4 Laboratory of Mass Spectrometry, Department of Chemistry, University of Liège, 4000 Liège, Belgium; loic.quinton@uliege.be

**Keywords:** cone snail toxins, conopeptide, α-conotoxin, drug development, electrophysiology, ion channel diseases, nicotinic acetylcholine receptor

## Abstract

The a-Conotoxins are peptide toxins that are found in the venom of marine cone snails and they are potent antagonists of various subtypes of nicotinic acetylcholine receptors (nAChRs). Because nAChRs have an important role in regulating transmitter release, cell excitability, and neuronal integration, nAChR dysfunctions have been implicated in a variety of severe pathologies. We describe the isolation and characterization of α-conotoxin MilIA, the first conopeptide from the venom of *Conus milneedwardsi.* The peptide was characterized by electrophysiological screening against several types of cloned nAChRs that were expressed in *Xenopus laevis* oocytes. MilIA, which is a member of the α3/5 family, is an antagonist of muscle type nAChRs with a high selectivity for muscle versus neuronal subtype nAChRs. Several analogues were designed and investigated for their activity in order to determine the key epitopes of MilIA. Native MilIA and analogues both showed activity at the fetal muscle type nAChR. Two single mutations (Met9 and Asn10) allowed for MilIA to strongly discriminate between the two types of muscle nAChRs. Moreover, one analogue, MilIA [∆1,M2R, M9G, N10K, H11K], displayed a remarkable enhanced potency when compared to native peptide. The key residues that are responsible for switching between muscle and neuronal nAChRs preference were elucidated. Interestingly, the same analogue showed a preference for α9α10 nAChRs among the neuronal types.

## 1. Introduction

Cone snails developed a powerful and efficient biological strategy to capture their prey and to defend against potential predators in order to survive. Cone snails are equipped with a sophisticated venom apparatus, wherein a cocktail of biologically active components is synthesized by epithelial cells lining the venom duct to overcome their relatively limited locomotory compared to other aquatic organisms [1]. Typically, the cocktail is made up of a complex subset of enzymes and peptides commonly referred to as conopeptides or conotoxins [2]. It is known that the venom of each individual species consists of 50 to 200 conotoxins [3]. These conotoxins are secreted by the venom gland and then injected into the prey via a radular tooth. Consequently, the conotoxins will spread into the bloodstream to find their way to target voltage-gated ion channels (e.g., sodium, potassium and calcium) or ligand gated ion channels (e.g., nicotinic acetylcholine receptor) that play a major role in the nervous system [4]. This results in a rapid prey immobilization, which is crucial for a successful hunting strategy. According to their feeding habits, cone snails are either fish hunting (piscivorous class), mollusk hunting (molluscivorous class), or worm hunting (vermivorous class) [1].

Like an extraordinary chemist, cone snails provide us with a goldmine of therapeutic peptides, the conotoxins. It is estimated that there are ca. 800 cone snail species [2,5]. This indicates that cone snails constitute a large library of potential neuropharmacological active compounds that are of great importance for the discovery of new therapeutics. However, only 0.2% of all cone snail peptides have been cataloged [6]. Despite the small number of conotoxins studied, already eight conotoxins demonstrated potential therapeutic effects in preclinical or clinical phase [1]. Moreover, in 2004, the United States Food and Drug Administration (FDA) approved the first conotoxin-derived drug, called Prialt. Prialt contains ziconotide, which is a synthetic equivalent of the natural conotoxin ω-MVIIA isolated from the piscivorous *Conus magus* [7]. This commercially available medicine is an analgesic and it is used to treat chronic pain in cancer and AIDS patients. Ziconotide inhibits N-type calcium channels that play a key role in the regulation of presynaptic neurotransmitter release [8]. However, Prialt is associated with some undesirable side effects at therapeutic dosage, like confusion, weakness, low blood pressure, and sleepiness. Interesting is the α-conotoxin Vc1.1, which selectively targets α_9_ α_10_-nAChRs and displays analgesic effects in rodent pain models with no reported side effects [9]. Vc1.1 was tested in human clinical trials and it successfully passed the safety tests in phase I. Unfortunately, due to the differential activity at human and rat α9α10-nAChRs, clinical trials were terminated after phase II [9].

Dysfunction of nAChRs is linked to several diseases, such as muscular dystrophies, Alzheimer’s disease, Parkinson’s disease, and pain [10,11,12,13]. Therefore, the discovery of new subtype specific ligands is important while studying the role of nAChRs in these neurodegenerative and psychiatric diseases. a-conotoxins are small peptides found in the venom of cone snails. Most a-conotoxins adopt a Cysteine Framework I scaffold, which is characterized by four Cys in the primary sequence and forming the pattern CC-C-C. The four cysteines form two disulfide bonds between Cys1-Cys3 and Cys2-Cys4, known as the globular disulfide connectivity. The peptide segments between Cys2 and Cys3 and between Cys 3 and Cys 4 are referred as loops, the length of which are used to sub-classify the a-conotoxins. Various subclasses of α-conotoxins have different receptor subtype selectivity. It is even believed that the pharmacological activity roughly correlates with the number of amino acids between the two loops of cysteine residues (m/n), where m and n represent the number of residues in the first and second loop, respectively [3,4]. The 3/5 conotoxins typically block muscle-type nAChRs, whereas 4/3, 4/4, 4/5, 4/6, and 4/7 classes typical interact with the neuronal-type nAChR [3,4].

In this work, we report the identification and characterization of a novel 3/5 α-conotoxin, MilIA from the venom of *Conus milneedwardsi.* Furthermore, several analogues of this peptide were synthesized and tested along with the native peptide for their inhibitory activity on various nAChR subunit compositions. Moreover, one analogue, MilIA [∆1,M2R, M9G, N10K, H11K], displayed a remarkable enhanced potency when compared to native peptide. The key residues that are responsible for switching between muscle and neuronal nAChRs preference were elucidated. Interestingly, the same analogue showed a preference for α9α10 nAChRs among the neuronal types.

## 2. Results

**Figure 1:**
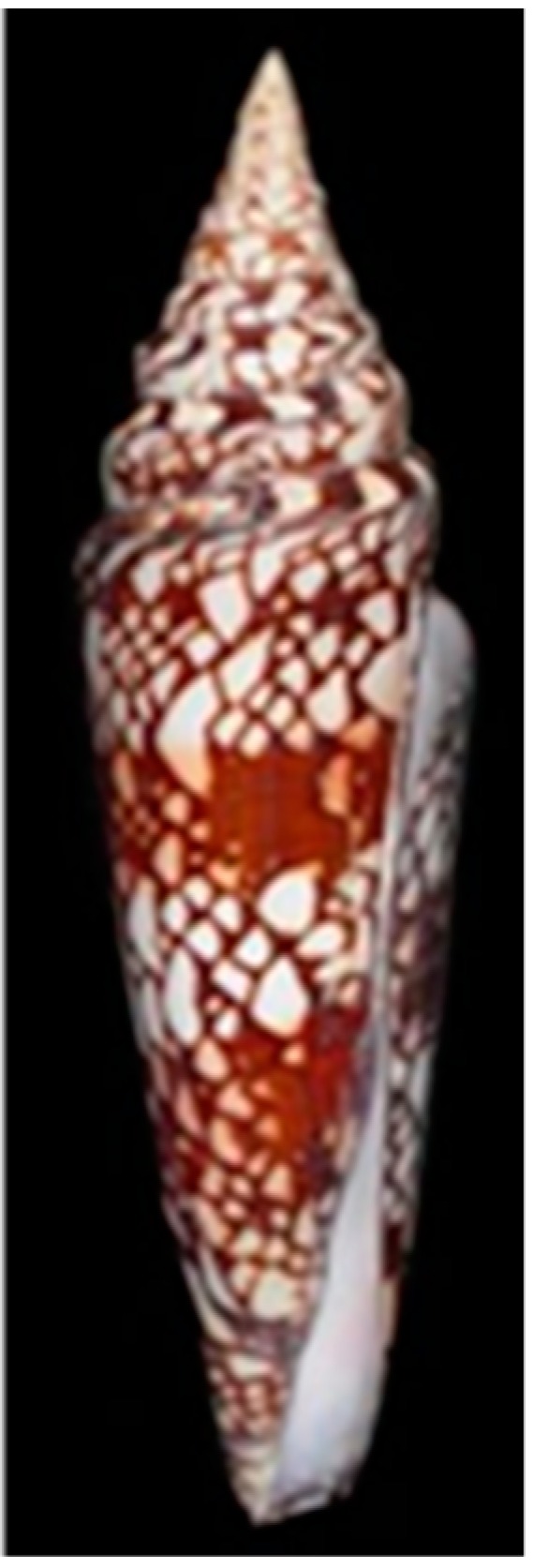
The shell of *Conus* *milneedwardsi*.

*Conus milneedwardsi* is also known as the glory of India, because of its remarkably beautiful shell (Figure 1). It is a mollusk hunting cone snail species commonly found in the Indian Ocean in Tamil Nadu, India. The venom of C. *milneedwardsi* has not yet been studied. To the best of our knowledge, no conotoxin sequences have been reported from this species so far [14].

### 2.1. Isolation and Identification of a Novel α-Conotoxin in C. milneedwardsi Venom

**Figure 2:**
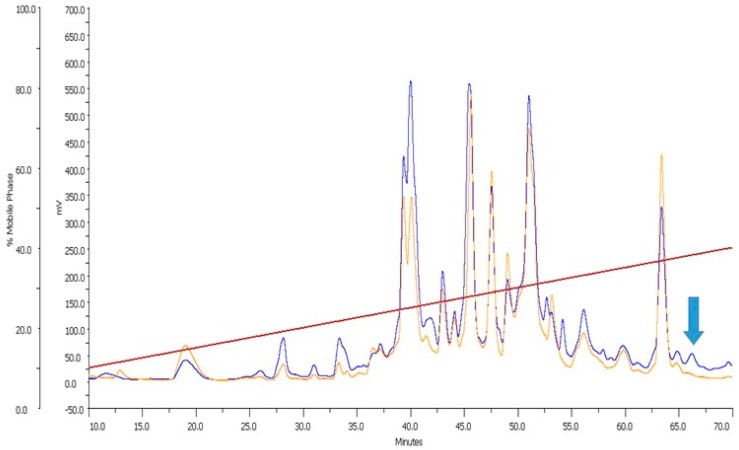
Crude venom of *C. milneedwardsi* was fractionated by C18 reversed-phase high-performance liquid chromatography (RP-HPLC). The absorbance was monitored at 280 nm (orange line) and at 214 nm (blue line). The red line represents the acetonitrile gradient. The fraction containing the peptide MilIA is indicated with a blue arrow.

The venom of *C. milneedwardsi* was subjected to reversed-phase high-performance liquid chromatography (RP-HPLC) purification (Figure 2). The initial screening of the venom fractions revealed promising activity on nAChRs. Further function-guided purification resulted in the identification of an active peptide. N-terminal Edman degradation of this peptide revealed a novel 14-residue α-conotoxin, called MilIA, with the sequence DMCCHPACMNHFNC and a molecular mass of 1620.85 Da, as determined by MALDI-TOF mass spectrometry (Figure 2), Supplemental Figures S1 and S2). This is in perfect agreement with the theoretically calculated mass of 1620.92 Da. To the best of our knowledge, MilIA is the first conotoxin that was isolated and pharmacologically characterized from *C. milneedwardsi*.

MilIA has three amino acids in the m loop and five in the n loop. This classifies the peptide as an α3/5 conotoxin. A sequence alignment shows that MilIA exerts high homology for the m loop with other conotoxins of the α3/5 family (Table 1). However, the residues in the n loop are less well conserved.

### 2.2. Functional Characterisation of MilIA and its Analogues

#### Activity of MilIA and its Analogues on Muscle-Type nAChRs

**Figure 3:**
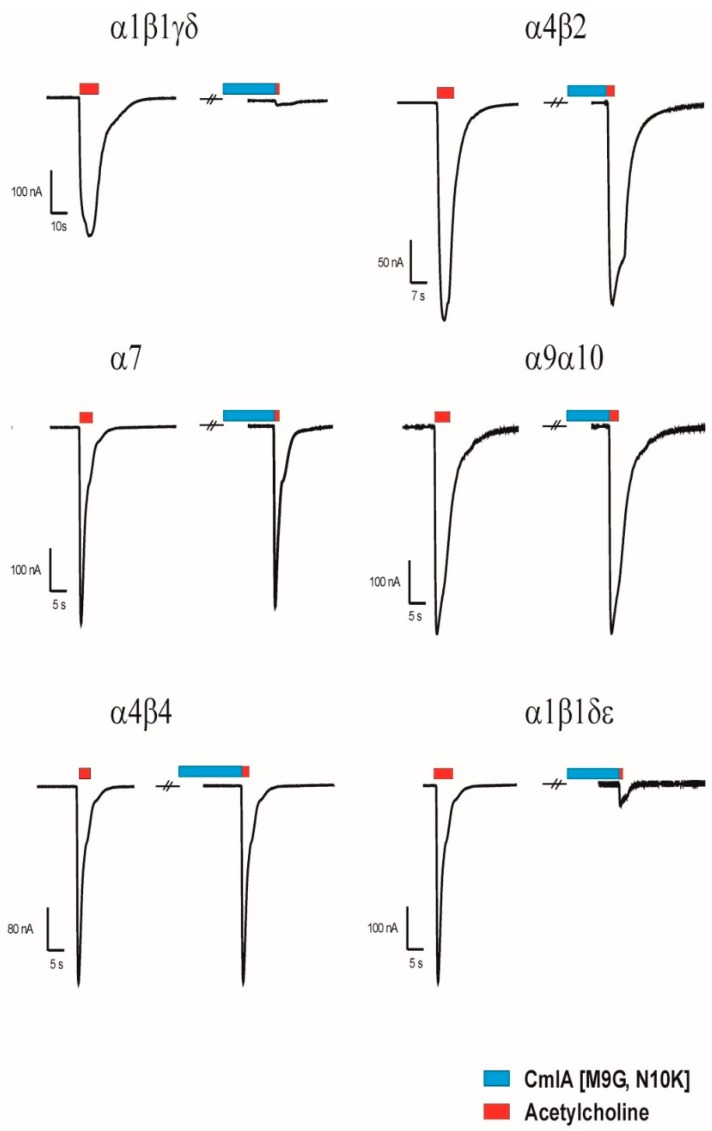
Electrophysiological profile of MilIA [M9G, N10K]. The electrophysiological profile showed the ACh-evoked current mediated by α1β1γδ, α4β2, α7, α9α10, α4β4, and α1β1δε nAChRs. The nAChRs were gated by a variable time duration pulse of ACh at 2 mL/min. (red) (200 µM for α_1_β_1_γδ, α4β2, α4β4, α1β1δε; 100 µM for α7; 500 µM for α9α10) for different nAChR subtypes. The first and the second peak amplitude represented the absence and presence of 1 µM of MilIA [M9G, N10K], respectively. The conopeptide was applied for 60 s at 2 mL/min. (blue), immediately followed by a variable time duration pulse of ACh (red).

Several members of the α3/5 conotoxins have been subjected to intesive structure-function studies. More specific for MI, GI an SI, the key residues have been previously investigated [4,15,16,17,18]. Based on these studies, we designed five MilIA analogous with specific residues mutated in order to investigate the importance of these amino acids on the activity of MilIA.To probe the degree of potency and selectivity, MilIA and its five analogous (MilIA [∆1,M2R], MilIA [M9G], MilIA [N10K], MilIA [M9G, N10K], and MilIA [∆1,M2R, M9G, N10K, H11K]) were tested for their inhibitory activity on the Ach-evoked current amplitude mediated by α_1_β_1_γδ, α_1_β_1_γε, α2β4, α4β2, α4β4, α7, and α9α10 nAChR. MilIA and analogues were screened at a concentration of 1 µM. There was no inhibition of any of the peptides on α2β4, α4β2, α4β4, and α7 (Table 2). However, MilIA, MilIA [∆1,M2R], MilIA [M9G], MilIA [N10K], and MilIA [M9G, N10K] elicited an inhibition of 20%, 10%, 60%, 25%, and 98%, respectively, of the ACh-evoked current amplitudes of the fetal muscle type α_1_β_1_γδ nAChR. For MilIA [M9G, N10K], the peptide with the highest activity, the electrophysiological profile, is shown in Figure 3. This electrophysiological profile demonstrated the capability of MilIA [M9G, N10K] to inhibit the ACh-evoked current amplitude at α_1_β_1_γδ nAChR for 98% and the incapability to exert an effect on the other subtypes (α4β2, α4β4, α7, and α9α10) tested. This result suggests an interesting target selectivity of MilIA [M9G], MilIA [N10K], and MilIA [M9G, N10K] for the fetal muscle type nAChR.

**Figure 4:**
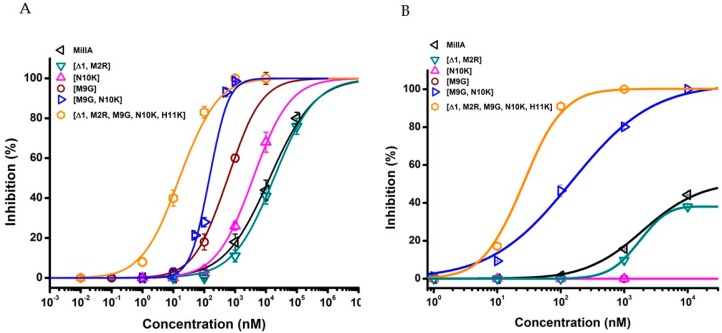
Concentration curves on α1β1γδ (**A**) and α1β1δε (**B**) nAChR. The percentage of inhibition of MilIA, MilIA [M9G], MilIA [N10K], MilIA [∆1,M2R], MilIA [M9G, N10K], and MilIA [∆1,M2R, M9G, N10K, H11K] at α1β1γδ (left) and α1β1δε (right) nAChR was plotted against the logarithm of the different concentrations tested and fitted with the Hill equation. The visualized error bars represent the standard error of the mean (S.E.M). All of the experiments were repeated at least three times (*n* ≥ 3).

In order to evaluate the potency of MilIA, MilIA [∆1,M2R], MilIA [M9G], MilIA [N10K], and MilIA [M9G, N10K], a concentration-response curve was plotted and the IC_50_ values were determined. The IC_50_ values yielded 13130 ± 1125 nM, 18012 ± 1430 nM, 577 ± 79 nM, 3842 ± 148 nM, and 178 ± 57 nM for MilIA, MilIA [∆1,M2R], MilIA [M9G], MilIA [N10K], and MilIA [M9G, N10K], respectively (Figure 4A, Table 2). The IC_50_ values of MilIA [∆1,M2R], MilIA [M9G], MilIA [N10K], and MilIA [M9G, N10K] decreased approximately two-fold, 23-fold, three-fold, and 74-fold, respectively, as compared to the native MilIA, indicating that the MilIA isoforms exhibited a higher potency when compared to the native MilIA. To compare the degree of potency and selectivity, MilIA, MilIA [M9G], MilIA [N10K], and MilIA [M9G, N10K] were also tested for their inhibitory activity on the ACh-evoked current amplitude mediated by adult muscle type α1β1δε nAChR. Interestingly, the single mutations [M9G] and [N10K] did not inhibit the adult muscle type nAChR in comparison with the increased potency obtained during the screening of fetal muscle type nAChR. This very rare characteristic indicated that both of the peptides were able to make a distinction between the two muscle nAChR subtypes. However, both of the peptides lose their capacity to distinguish between adult and fetal muscle type nAChR when both mutations were combined. The differences in potency of MilIA [M9G, N10K] between the two muscle types was minimal. The IC_50_ values of MilIA [M9G, N10K] decreased by approximately 75-fold for both the fetal and the adult muscle type nAChR, relative to the native peptide. Concentration-response curves were obtained and IC_50_ values were determined in order to evaluate the potency of MilIA and its analogues on the fetal nAChR (Figure 4B, Table 2).

**Figure 5:**
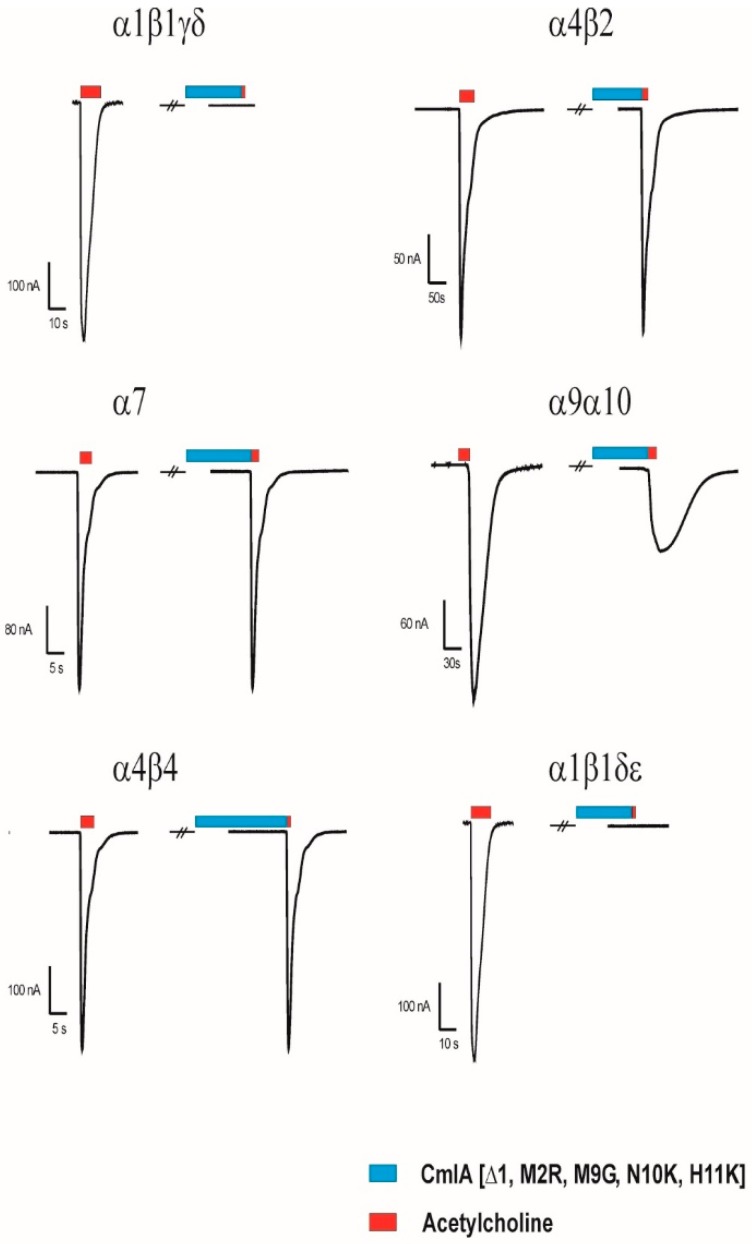
Electrophysiological profile of MilIA [∆1,M2R, M9G, N10K, H11K]. The electrophysiological profile showed the ACh-evoked current mediated by α1β1γδ, α4β2, α7, α9α10, α4β4 and α1β1δε nAChRs. The nAChRs were gated by a variable time duration pulse of ACh (red) (200 µM for α1β1γδ, α4β2, α4β4, α1β1δε; 100 µM for α7; 500 µM for α9α10) for the different nAChR subtypes at 2 mL/min. The first and the second peak amplitude represented the absence and presence of 1 µM of MilIA [∆1,M2R, M9G, N10K, H11K], respectively. The conopeptide was applied for 60 s at 2 mL/min. (blue), immediately followed by the addition of a variable time duration pulse of ACh (red).

In Figure 5, the activity profile of MilIA [∆1,M2R, M9G, N10K, H11K] at a concentration of 1 µM is shown. It was observed that this analogue exerted affinity for both the muscle type α1β1γδ nAChR (100% inhibition) and the neuronal subtype α9α10 nAChRs (73% inhibition). However, no effect on the Ach-evoked current amplitudes that were mediated by other neuron nAChR subtypes (α4β2, α4β4, α2β4, and α7) was observed (Figure 5, Table 2). Thus, MilIA [∆1,M2R, M9G, N10K, H11K] is able to target a specific neuronal subunit of nAChR (α9α10) without affecting the other neuronal subytpes (α4β2, α4β4, α2β4, and α7) nAChRs. Interestingly, MilIA [∆1,M2R, M9G, N10K, H11K] demonstrated the biggest shift to the left, which proved the highest potency towards the fetal muscle type α1β1γδ nAChR as compared to the other mutants synthesized (Figure 4B). The IC_50_ value of MilIA [∆1,M2R, M9G, N10K, H11K] yielded 16 ± 1 nM. Thus, a decrease of approximate 820-fold in IC_50_ value for MilIA [∆1,M2R, M9G, N10K, H11K] when compared to the native MilIA was observed.

**Figure 6:**
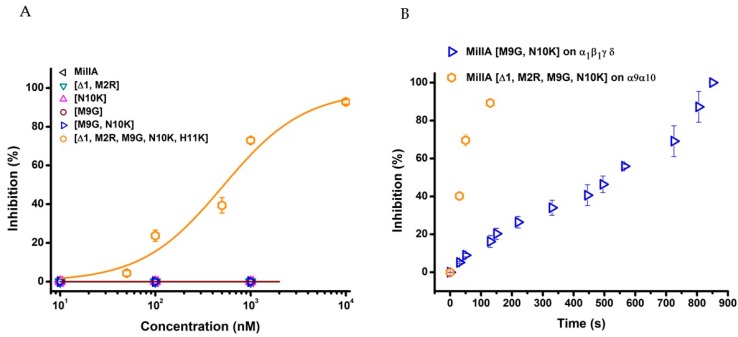
(**A**) Concentration-response curves on α9α10 nAChR. The percentage of inhibition was plotted against the logarithm of the different concentrations tested and fitted with the Hill equation. The visualized error bars represent the standard error of the mean (S.E.M). All experiments were repeated at least three times (*n* ≥ 3). (**B**) The time duration of the recovery of nAChR response to ACh after inhibition. Blue line: washout kinetic for MilIA [M9G, N10K] on α1β1γδ nAChR; Orange line: washout kinetic for MilIA [∆1,M2R, M9G, N10K, H11K] on α9α10 nAChR.

MilIA [∆1,M2R, M9G, N10K, H11K] was the only analogue that was tested that had the ability to inhibit the neuron type α9α10 nAChRs. The IC_50_ value yielded 511 ± 195 nM (Figure 6A).

**Figure 7:**
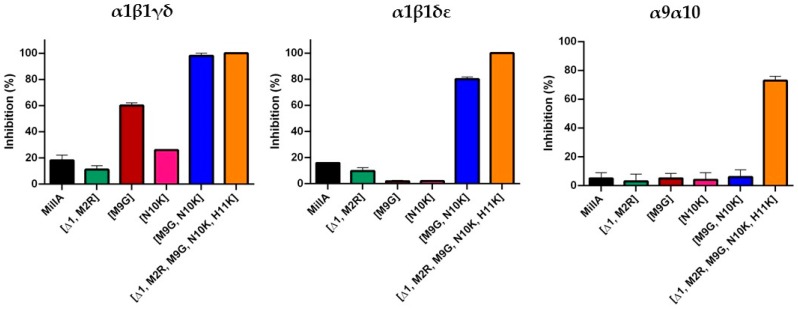
Percentage inhibition of the Ach response after addition of 1 µM MilIA.

To test the reversibility actions of MilIA [M9G, N10K] on α_1_β_1_γδ nAChR and MilIA [∆1,M2R, M9G, N10K, H11K] on α9α10 nAChR, the time duration of the recovery of nAChR response to ACh was examined. During the experiments, the oocytes expressing the α_1_β_1_γδ nAChR subtype were continuously perfused with a ND96 solution. A control response was obtained by adding 200 µM ACh. Afterwards, 1 µM of MilIA [M9G, N10K] was added for 200 s, directly followed by a 2 s impulse of ACh. Subsequently, a washout period with ND96 solution followed while responses to ACh were recorded. The same experimental procedure was performed for MilIA [∆1,M2R, M9G, N10K, H11K] (500 µM ACh) on α9α10 nAChR. Figure 6B shows the time course for MilIA [M9G, N10K] and for MilIA [∆1,M2R, M9G, N10K, H11K]. The α9α10 nAChR recovered very fast, and full recovery was achieved after 2 min. washing. The recovery of α_1_β_1_γδ nAChR was much slower and total recovery was achieved after 14 min. Figure 7 shows the activity profile of MilIA and all analogous on the muscle type and α9α10 isoforms.

## 3. Discussion

Cone snails, in particular, are seen as the new medicinal chemist that has the capacity to perform complex peptide chemistry to make an arsenal of selective therapeutic toxins. Druggable toxins are more and more investigated as future generation drugs because of their mode of action towards ion channels. A defect of ion channels like nAChR are often related with diseases, such as cancer, pain, neuromuscular defects, multiple sclerosis, and Alzheimer’s disease. α-Conotoxins have long been associated with both muscle type and neuronal type nAChRs and they are able to antagonize it, which is one step forward in the treatment of these diseases. Today, only a narrow range of potential lead compounds from the large library of neuropharmacological active toxins produced by cone snails is invested.

The present study presents the identification and functional characterization of a novel α-conotoxin, called MilIA, from *Conus milneedwardsi.* Synthetic analogues were generated to find the key epitopes of this peptide and to see whether it would be possible to improve the target specificity and potency towards the receptor subtype of interest. MilIA exhibits a typical 3/5 framework. It is well described that α-conotoxins displaying a 3/5 framework preferentially target the muscle type nAChR [19]. Thus it was not surprising that the native MilIA showed affinity towards the muscle type nAChR. Depending on the stage of development, fetal or adult, the muscle cells express nAChRs composed of α_1_β_1_γδ or α_1_β_1_εδ subunits, respectively. The primary binding places of the antagonist are positioned at the interface of α_1_δ and α_1_γ(ε) [11,19].

**Figure 8:**
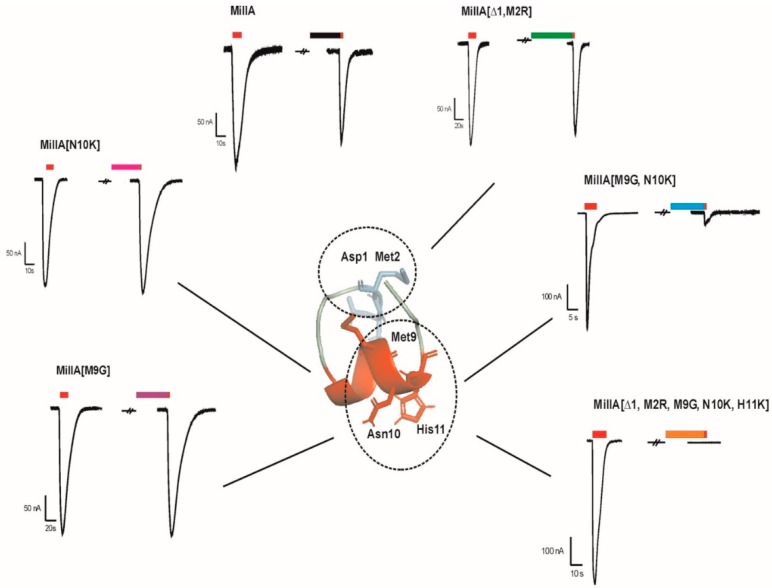
Selected traces of the α1β1δε nAChR showing effects induced by MilIA and its mutants. All of the components tested at a concentration of 1 µM could induce a block. The double and triple mutants showed the highest inhibition. The visualized error bars represent the standard error of the mean (S.E.M). All experiments were repeated at least three times (*n* ≥ 3). The key residues are highlighted as lines.

Among the α3/5 conotoxins, α-conotoxins MI, GI, and SI are the three most investigated peptides. The IC_50_ values of these conotoxins range from 20–40 nM, with an exception for α-conotoxin MI with an IC_50_ value of 0.4 nM [20]. Remarkably different from this data, was that native α-conotoxin MilIA showed a low potency, towards the muscle type nAChR (IC_50 fetal_ = 13,130 ± 1125 nM and IC_50 adult_ = 1118 ± 78,891 nM). Therefore, synthetically produced analogues of MilIA were studied to expose the key residues important for selectivity on a nAChR subtype. Generally, it is known that the residues in loop 2 are responsible for the selectivity, while the residues in loop 1 are crucial for binding [4,19]. Two analogues, MilIA [M9G] and MilIA [N10K], had a selective activity at the human fetal muscle type α_1_β_1_γδ nAChR without affecting the neuronal nAChR subtypes. An overview the activity of MilIA and its mutants on the α1β1δε nAChR is provided in Figure 8. Additionally, the potency of MilIA [M9G] and MilIA [N10K], increased 23-fold and three-fold, respectively. These findings suggest that both position 9 and position 10 play an important role in the interaction with α_1_β_1_γδ nAChR. It even revealed that a much higher inhibitory potency can be achieved by introducing a Gly at position 9.

As can be seen in Figure 3, most α3/5 conotoxins, which also display a higher potency towards muscle type nAChRs, have a Gly residue immediately following the third Cys residue. Previous studies have highlighted the importance of this glycine and its function for the selectivity at the fetal muscle type nAChR [21,22]. The conotoxin MI was used to investigate the binding interactions between the conotoxin and α/δ binding site on the nAChR [16]. A strong hydrophobic interaction was seen between the Pro at position 6 and the Gly at position 9 and the residues on the α/δ binding site [15]. These interactions appear to stabilize the binding. It has been shown that the small size and angle of Gly at position 9 is determining for the high affinity [19,23]. More recently, it was shown for toxin GI that the substitution of a Gly for an Ala reduced the affinity for the α/δ binding site by approximately 30-fold [24]. It has been suggested that the Gly has a significant impact on the right-handed helical turn from the α-helix. Presumably, the above statements can also explain the increase in potency, following the substitution of Met for a Gly in MilIA.

The improved potency of MilIA [N10K] at the fetal muscle type nAChR was in agreement with previous studies, which investigated the affinities of different isoforms of the α-conotoxins SI. In SI, substitution of a Pro for a positive charged Lys at a corresponding position, as Asn in MilIA resulted in a 190-fold increase affinity for the α_1_γ binding site of *Torpedo* nAChR. The increased potency was explained by an alteration of the backbone structure at this position. This residue is oriented towards the binding subunits γ and δ, making subunit dependent contacts [25]. Similar, the introduction of a Lys residue at this position in MI also increased the affinity at the *Torpedo* nAChR [26]. A negative charge at position 10 drastically decreases the affinity of MI. Additional studies also showed that, for MI, the presence of a cationic residue at position 10 allows for a strong π-cation interaction with the aromatic residues in the binding site γY111 over the δArg-113 of the *Torpedo* nAChR [27]. Interestingly, combining the Gly9 and the Lys10 mutations, resulted in a synthetic variant with 75-fold higher potency at the fetal muscle type relative to the native MilIA peptide.

The same synthetic analogues were also tested on the adult muscle type nAChR. Native MilIA and most of the analogues showed a similar affinity for fetal and adult muscle nAChRs. However, two MilIA analogues are able to discriminate between adult and fetal muscle type nAChR compositions. The peptides MilIA [M9G] and MilIA [N10K] have an approximately 580-fold and 3800-fold, respectively, higher affinity for the human fetal muscle type nAChR relative to the adult muscle type nAChR. Other conotoxins that are able to distinguish between fetal and adult muscle type nAChR are rather rare. αA-conotoxin OIVB, isolated from the venom of *C. obscurus*, exhibits an 1800-fold higher affinity for the human fetal muscle nAChR relative to the human adult nAChR [28]. ψ-conotoxin PrIIIE, from *Conus parius*, also shows preferential affinity for the adult subtype over the fetal subtype [18]. Interestingly, [M9G] and [N10K] mutations did not individually show affinity on the adult composition of muscle nAChRs. However, the peptide combining both mutations acquires a very high affinity for the adult muscle type. As a result, this peptide lost its capacity to distinguish between the adult and fetal subtype.

The removal of the N-terminal Asp and Met and the insertion of an Arg at position 0 of MilIA converted this peptide into a selective ligand for the muscle type, but with a two-fold decreased potency. This result is contrasting with previous work, showing that the removal of the Gly at the N-terminal of conotoxin MI resulted in an increase of potency for the fetal muscle type nAChR [29]. Nevertheless, structure-function studies with other α-conotoxins have indicated that the length of the N-terminus sequence is important for the binding to nAChRs [17,30]. Consequently, the decrease in potency of MilIA [∆1,M2R] might be explained by this importance of the length of the N-terminus.

MilIA [∆1,M2R, M9G, N10K, H11K] turned out to be the most potent analogue against the fetal muscle type nAChR. At position 11 of MilIA, a neutral charged His was replaced by a positively charged Lys. The effect of this substitution has already been investigated on conotoxin MI. The second loop of the synthetic variant of MI consisted out of a Gly, Lys and Lys at position 9, 10, 11, respectively. Previously, it was described that the Gly and Lys at position 9 and 10, respectively, are both important factors that determine the high potency within nanomolar range of conotoxin MI. The introduction of an extra positive charge (Lys) at position 11 only increased this potency slightly [26]. This is in accordance with the activity that was observed for MilIA [∆1,M2R, M9G, N10K, H11K]. Notably, the decreased potency that was caused by the [∆1,M2R] mutation disappeared. Notwithstanding these observations, more research is necessary to unravel the mode of action and pinpoint the exact binding sites of MilIA on nAChRs.

Among the neuronal nAChRs, MilIA [∆1,M2R, M9G, N10K, H11K] only affected α9α10 nAChR without showing affinity for the other neuronal type nAChR subtypes tested (α4β2, α4β4, α2β4, and α7). MilIA [∆1,M2R, M9G, N10K, H11K] was the only peptide that was investigated in this study that showed activity at a neuronal subtype of nAChR. This target specificity is rather unusual, since α3/5 conotoxins are reported to be mainly muscle type nAChRs antagonist. To the best of our knowledge, no α-conotoxins with a 3/5 framework targeting the α9α10 nAChR has been reported. So far, only a few examples of α-conotoxins that target both muscle type nAChR and neuronal α9α10 nAChR are described in the literature [11,31]. For instance, αB-VxXXIVA from *C. vexillum* inhibits muscle type nAChR and α9α10 nAChR with micromolar potency (IC_50_ of 6.6 µM and 1.2 µM, respectively). α-O-GeXVIA from *C. generalis* inhibited the muscle nAChR and α9α10 nAChR with nanomolar potency. However, it should be noted that both of these conotoxins also target several other subtypes of the neuronal nAChR and thus lack specificity.

To date, Vc1.1 (α4/7 conotoxin) and RgIA (α4/3 conotoxin) are the two most promising α-conotoxins, selectively inhibiting the α9α10 nAChR [8]. Previously, scanning experiments were done to reveal the key residues of these conotoxins for their interaction with the α9α10 receptor [11]. For RgIA, it was found that Asp5, Pro6, Arg 7, and Arg9 were crucial for the selectivity towards α9α10 [32]. Similar mode of actions was found for Vc1.1. A loss of activity at α9α10 nAChR was seen when Asp5 and Asp11, Arg7, and Ile15 were changed [9]. Replacing the Asn9 turns out to be important in increasing the potency of Vc1.1. When position 9 of Vc1.1 contained a hydrophobic residue, two smaller hydrophobic regions are joined to form a hydrophobic patch, which leads to an increased potency [33]. The same increase in potency was seen when a small non-hydrophobic residue, Gly, was inserted at position 9. Consequently, the Gly act as a small, inert amino acid space filler that does not affect configuration, which indicates that the size of the residue is important for an increase in potency. Unfortunately, due to low sequence homology, it is difficult to make relevant conclusions regarding possible key residues for MilIA [∆1,M2R, M9G, N10K, H11K] interaction with the α9α10 receptor.

Great progress has also been made in the last few years to get insight into the neuronal type α9α10 nAChRs. The discovery of the antagonists RgIA and Vc1.1, significantly aided in the identification of α9α10 nAChRs as a molecular target in the treatment of neuropathic pain [11,31,34]. A recent study demonstrated the use of αO-Conotoxin GeXIVA as a new analgesic drug to treat chemotherapy-induced neuropathic pain in a rat model that could bypass the side effects of the current used chemotherapeutic, oxaliplatin [30]. The same conotoxin was also investigated in a study regarding cervical cancer. It is known that a dysfunction of nAChRs is related with the tumor development, such as tumor growth, angiogenesis, metastasis, etc. [35]. A regulation of nAChRs can lead to the discovery of new anticancer drugs. More specifically, it was found that α9 and α10 are over expressed in SiHA and CaSki cells. Treatment with αO-Conotoxin GeXIVA inhibited the proliferation of cancer cells and it suggests potential use for cervical cancer targeted therapy [31]. A peptide, such as MilIA [∆1,M2R, M9G, N10K, H11K], with strong selectivity towards α9α10 nAChR, could therefore be interesting for the development of new analgesic or anticancer drug. However, potential use of an α9α10 nAChR targeting drug will require the modification of the peptide in order to eliminate its affinity for muscle type nAChRs.

## 4. Materials and Methods

### 4.1. Crude Venom Extraction from Conus milneedwardsi

All of the specimens of *C. milneedwardsi* used in this study were collected in the Indo-Pacific Ocean, near Tamil Nadu in India. The venom gland was dissected and then preserved in RNAlater™ solution at −20 °C for analysis at a later date. An extraction protocol was performed to isolate the peptides from the venom gland of *C. milneedwardsi*. Venom gland was first thawed on ice. Afterwards, it was crushed with a mortar and 4 mL of 50% acetonitrile (ACN, Biosolve BV., the Nederlands) was added before it was stored at −80 °C. The pulverized venom glands were then transferred into a beaker with 50% ACN and then stirred overnight. The supernatant was freeze dried and stored at −20 °C. The venoms extracted from the glands of seven specimens were pooled together.

### 4.2. Peptide Purification

Peptide purification and folding were assessed by analytical reversed-phase high-performance liquid chromatography (RP-HPLC) while using a Vydac C18 column (Grace Davison Discovery Sciences, Aiken, SC, USA) and a dual wavelength absorbance detector (UV/VIS-155 detector; Gilson, Middleton, WI, USA). During the analysis, fractions were eluted with a linear gradient from 0% to 80% of solvent B (0.1% TFA (*v*/*v*) in 90% aqueous acetonitrile) over 90 min. at a flow rate of 1 mL/min.

### 4.3. Peptide Sequencing

Purified peptide was subjected to a N-terminal amino acid sequence analysis that was performed by an automatic protein sequencer (Protein Sequencer PPSQ-31A/33A; Shimadzu, Kyoto, Japan).

### 4.4. Mass Spectrometry Analysis

Mass spectrometry experiments have been performed on a MALDI-TOF/TOF spectrometer (Rapiflex, Bruker Daltonics), externally calibrated from *m/z* 757 to 3150 while using PepMix Calibration Mixture II (Bruker). Dried venom fractions were dissolved in 200 µL of formic acid (0.1%), vortexed ten seconds, and then centrifuged. 1 µL of each solution was deposited on a MALDI plate together with 1 µL of saturated CHCA (80% ACN/20% FA 0.1%). The droplets were let to dry at room temperature to co-crystallize the matrix and the peptides. Laser shots at 38% irradiated the crystals, and 2000 spectra were accumulated to obtain a satisfying signal to noise ratio.

### 4.5. Peptide Synthesis and Folding

Wild-type MilIA and its analogous were designed while using Fmoc (N-(9-fluorenyl)methoxycarbonyl) solid-phase peptide synthesis chemistry by GenicBio Limited (Shanghai, China). 10 mg of each peptide was synthesized. The synthesized peptides were lyophilized and kept at room temperature (18–22 °C). The peptides were folded by dissolving them in a physiological buffer solution ND96, 2 mM KCl, 2 mM MgCl2, 1.8 mM CaCl2, and 5 mM HEPES at a concentration of 1 mg/mL. The folding and purity were analyzed with MALDI-TOF (Supplemental Figures S1 and S2).

### 4.6. Functional Characterization

#### 4.6.1. Expression of Voltage-Gated ion Channels in Xenopus Laevis Oocytes

For the expression of human AChR (α1, α2, α3, α4, α7, α9, α10, β2, β4, hγ; hδ; hε) in Xenopus oocytes, the linearized plasmids were transcribed while using the T7 or SP6 mMESSAGE-mMACHINE transcription kit (Ambion^®^, Carlsbad, CA, USA). The harvesting of stage V–VI oocytes from anaesthetized female Xenopus laevis frog was previously described [36]. Oocytes were injected with 50 nL of cRNA at a concentration of 1 ng nL − 1 using a micro-injector (Drummond Scientific^®^, Broomall, PA, USA). The oocytes were incubated in a solution containing (in mM): NaCl, 96; KCl, 2; CaCl_2_, 1.8; MgCl_2_, 2; and, HEPES, 5 (pH 7.4), supplemented with 50 mg L − 1 gentamycin sulfate.

#### 4.6.2. Electrophysiological Recordings

Two-electrode voltage-clamp recordings were performed at room temperature (18–22 °C) while using a Geneclamp 500 amplifier (Molecular Devices^®^, Downingtown, PA, USA) controlled by a pClamp data acquisition system (Axon Instruments^®^, Union City, CA, USA). Whole cell currents from oocytes were recorded 1–4 days after injection. Bath solution composition was (in mM): NaCl, 96; KCl, 2; CaCl_2_, 1.8; MgCl_2_, 2; and, HEPES, 5 (pH 7.4). Voltage and current electrodes were filled with 3 M KCl. Resistances of both electrodes were kept between 0.7 and 1.5 MΩ. During recordings, the oocytes were voltage-clamped at a holding potential of −70 mV and continuously super fused with ND96 buffer via gravity-fed tubes at 0.1–0.2 mL min^−1^, with 5 min. incubation times for the bath-applied peptides. Acetylcholine (ACh) was applied via gravity-fed tubes until peak current amplitude was obtained (1–3 s), with 1–2 min. washout periods between applications. The nAChRs were gated by a variable time duration pulse of ACh (200 µM for α1β1γδ, α4β2, α4β4, α1β1δε; 100 µM for α7; and, 500 µM for α9α10) for the different nAChR subtypes at 2 mL/min. Data was sampled at 500 Hz and filtered at 200 Hz. Peak current amplitude was measured prior to and following the incubation of the peptide.

To assess the concentration-response relationships, data points were fitted with the Hill equation: y = 100/[1 + (EC50/[toxin])h], where y is the amplitude of the toxin-induced effect, EC50 is the toxin concentration at half maximal efficacy, [toxin] is the toxin concentration and h is the Hill coefficient. A comparison of two sample means was made using a paired Student’s *t* test (*p* < 0.05). The data are presented as mean ± standard error (SEM) of at least six independent experiments (*n* ≥ 6). All the data were tested for normality using a D’Agostino-Pearson omnibus test. All the data were tested for variance while using Bonferroni test or Dunn’s test. Data following a Gaussian distribution were analyzed for significance while using one-way ANOVA. Non-parametric data were analyzed for significance using the Kruskal–Wallis test. Differences were considered to be significant if the probability that their difference stemmed from chance was <5% (*p* < 0.05). All data was analyzed while using pClamp Clampfit 10.0 (Molecular Devices^®^, Downingtown, PA, USA) and Origin 7.5 software (Originlab^®^, Northampton, MA, USA).

## 5. Conclusions

In summary, it was shown that mutations at position 9 and position 10 together are important in increasing the potency of MilIA for nAChRs. Residues at both positions turns out to be the key residues that are involved in the selective interaction of MilIA with muscle type nAChRs. The peptides MilIA [M9G] and MilIA [N10K] are capable of distinguishing between fetal and adult muscle type nAChR. The peptide MilIA [∆1,M2R, M9G, N10K, H11K] changed its target specificity towards both muscle type nAChR and neuronal type α9α10 nAChR. Moreover, among neuronal nAChR subtypes tested, this peptide selectively interacts with α9α10 nAChR. This study suggests MilIA as a potential interesting lead for further development and it shows that marine toxins are interesting compounds in the search for treatments for ion channels that are related diseases, such as cancer, pain, and muscle diseases [11,31].

## Figures and Tables

**Table 1:** Sequence comparison of α3/5 conotoxins with MilIA. The conserved cysteine residues colored in grey, C-terminal amide indicated with an asterisk and applied point mutations in red. The data was obtained via ConoServer database, aligned with ClustalX2.1. ∆1,M2R indicates a deletion of the first 2 N-terminal residues combined with a Met to Arg mutation at position 2.

CmIA	-DMCCH-PACMNHFN--C*	
CmIA [M9G]	-DMCCH-PACGNHFN--C*	
CmIA [N10K]	-DMCCH-PACMKHFN--C*	
CmIA [∆1,M2R]	--RCCH-PACMNHFN--C*	
CmIA [M9G,N10K]	-DMCCH-PACGKHFN--C*	
CmIA [∆1,M2R,M9G,N10K,H11K]	--RCCH-PACGKKFN--C*	
GI	--ECCN-PACGRHYS--C*	
MI	-GRCCH-PACGKNYS--C*	
SI	--ICCN-PACGPKYS--C*	
RgIA	--GCCSDPRCRYR----CR	
Vc1.1	--GCCSDPRCNYSHPEIC*	
MII	--GCCSNPVCHLEHSNLC*	
PIA	RDPCCSNPVCTVHNPQIC*	
LsIA	-SGCCSNPACRVNNPNIC*	

**Table 2:** IC_50_ values of MilIA and isoforms acting on different nAChR subtypes. The corresponding Hill coefficients are summarized in Table S1.

IC_50_	α1β1δε (nM)	α1β1γδ (nM)	α2β4 (nM)	α4β2 (nM)	α4β4 (nM)	α7 (nM)	α9α10 (nM)
MilIA	11,180 ± 78,891	13,130 ± 1125	>10^4^	>10^4^	>10^4^	>10^4^	>10^4^
MilIA [M9G]	>10^5^	577 ± 79	>10^4^	>10^4^	>10^4^	>10^4^	>10^4^
MilIA [N10K]	>10^5^	3842 ± 148	>10^4^	>10^4^	>10^4^	>10^4^	>10^4^
MilIA [∆1,M2R]	19,550 ± 50	18,012 ± 1430	>10^4^	>10^4^	>10^4^	>10^4^	>10^4^
MilIA [∆1,M2R, M9G, N10K, H11K]	25 ± 1	16 ± 1	>10^4^	>10^4^	>10^4^	>10^4^	511 ± 195
MilIA [M9G, N10K]	149 ± 39	178 ± 57	>10^4^	>10^4^	>10^4^	>10^4^	>10^4^

## Supplementary Materials

The following are available online at https://www.mdpi.com/1660-3397/17/9/535/s1, Figure S1: MALDI-TOF analysis of synthetic MilIA, Figure S2: Sequence verification of synthetic reduced MilIA, Table S1: Hill coefficient values for the obtained concentration-response curves.
