# Supplementary material for: Structure-Function Elucidation of a New α-Conotoxin, MilIA, from Conus milneedwardsi
Source: Mar Drugs. 2019 Sep 16;17(9):535. doi: 10.3390/md17090535 (PMC6780063; doi:10.3390/md17090535)
Supplement: Supplementary file 1 [file marinedrugs-17-00535-s001.pdf]

Table S1. Hill coefficient values for the obtained concentration-response curves.

Hill Coëfficiënt	α1β1δε (nM)	α1β1γδ (nM)	α9α10 (nM)
**MilIA**	1.2 ± 0.1	0.7 ± 0.1	/
**MilIA[M9G]**	/	1.0 ± 0.1	/
**MilIA[N10K]**	/	0.8 ± 0.1	/
**MilIA[Δ1,M2R]**	0.75 ± 0.1	0.7 ± 0.1	/
**MilIA[Δ1,M2R, M9G, N10K, H11K]**	0.9 ± 0.3	1.4 ± 0.4	1.03 ± 0.3
**MilIA[M9G, N10K]**	1.7 ± 0.1	1.8 ± 0.4	/

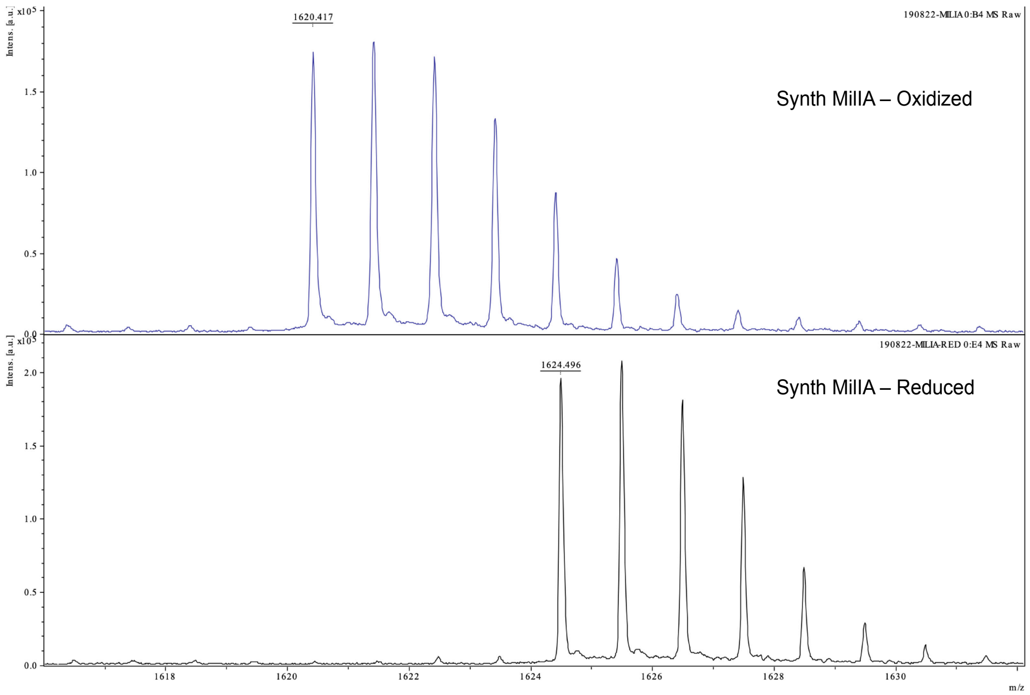

Figure S1. MALDI-TOF analysis of synthetic MilIA.

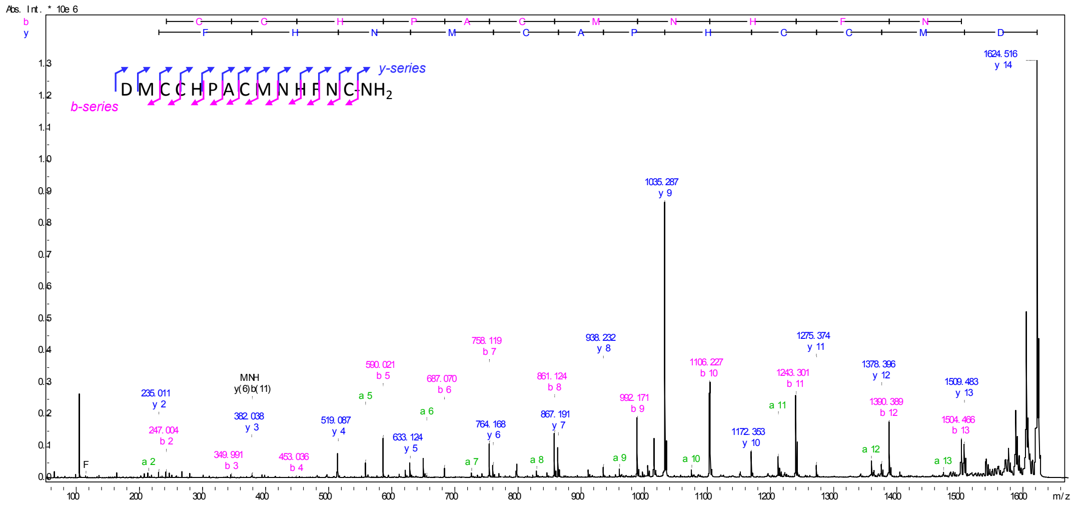

Figure S2. Sequence verification of synthetic reduced MilIA.
